# Supplementary material for: The Impact of Tea Consumption on Prediabetes Regression and Progression: A Prospective Cohort Study
Source: Nutrients. 2025 Jul 19;17(14):2366. doi: 10.3390/nu17142366 (PMC12298866; doi:10.3390/nu17142366)
Supplement: Supplementary file 1 [file nutrients-17-02366-s001.zip › nutrients-3738504-supplementary.pdf]

Supplementary Table S1. Baseline characteristics of participants with different types of tea.

	No tea （N=1198）	Green tea （N=1016）	Black tea （N=138）	Dark tea （N=140）	Other tea （N=170）	**P** overall
Gender						< 0.001
Male (%)	282 (23.5%)	509 (50.1%)	58 (42.0%)	41 (29.3%)	66 (38.8%)	
Female (%)	916 (76.5%)	507 (49.9%)	80 (58.0%)	99 (70.7%)	104 (61.2%)	
Age (y)	54.8 ± 8.4	53.8 ± 7.8**	51.8 ± 9.8***	51.0 ± 11.1***	49.8 ± 9.1***	< 0.001
BMI (kg/m2)	25.1 ± 4.4	25.7 ± 3.8***	27.1 ± 4.2***	27.7 ± 4.6***	25.7 ± 5.4	0.001
WC (cm)	84.2 ± 9.6	85.7 ± 10.1***	90.8 ± 11.1***	89.1 ± 12.3***	86.0 ± 9.9**	< 0.001
SBP (mmHg)	137.0 ± 20.0	137.1 ± 19.1	132.9 ± 20.5	136.0 ± 21.3	136.8 ± 20.4	0.26
DBP (mmHg)	83.0 ± 11.5	83.8 ± 11.4	81.8 ± 11.6	83.7 ± 13.1	84.5 ± 10.9	0.14
FPG (mmol/L)	5.9 ± 0.5	5.9 ± 0.4*	5.6 ± 0.6***	5.4 ± 0.6***	5.9 ± 0.4	< 0.001
2hPG (mmol/L)	7.3 ± 1.7	7.3 ± 1.7	7.0 ± 1.6	7.1 ± 1.8	7.1 ± 1.7	0.59
HbA1c (%)	5.5 ± 0.4	5.5 ± 0.4	5.6 ± 0.4	5.6 ± 0.4	5.4 ± 0.4	0.06
TC (mmo/L)	5.0 ± 1.0	5.0 ± 1.0	5.0 ± 0.9	5.2 ± 1.1	5.0 ± 1.0	0.47
TG (mmo/L)	1.8 ± 1.7	2.0 ± 2.3*	1.5 ± 0.7	1.5 ± 1.0	2.0 ± 2.4	< 0.001
HDL-C (mmo/L)	1.6 ± 0.4	1.5 ± 0.4	1.4 ± 0.3	1.5 ± 0.4	1.5 ± 0.4	0.29
LDL-C (mmo/L)	2.8 ± 0.8	2.7 ± 0.7	3.0 ± 0.7	3.1 ± 0.8	2.7 ± 0.7	0.28
eGFR (mL/min/1.73 m2)	98.3 ± 14.0	98.1 ± 13.6	95.0 ± 16.6*	97.5 ± 16.1	101.2 ±16.1*	< 0.001
Ethnicity, n (%)						< 0.001
Han	1024 (85.5%)	954 (93.9%)	40 (29.0%)	10 (7.1%)	149 (87.6%)	
Other	174 (14.5%)	62 (6.1%)	98 (71.0%)	130 (92.9%)	21 (12.4%)	
Vegetable consumption, n (%)						< 0.001
Minimal	4 (0.3%)	5 (0.5%)	0 (0.0%)	1 (0.7%)	0 (0.0%)	
Low	54 (4.5%)	46 (4.5%)	3 (2.2%)	10 (7.1%)	8 (4.7%)	
Moderate	602 (50.3%)	573 (56.4%)	99 (71.7%)	99 (70.7%)	95 (55.9%)	
High	538 (44.9%)	392 (38.6%)	36 (26.1%)	30 (21.4%)	67 (39.4%)	
Fruit consumption, n (%)						< 0.001
Minimal	39 (3.3%)	36 (3.5%)	3 (2.2%)	4 (2.9%)	6 (3.5%)	
Low	519 (43.4%)	529 (52.1%)	36 (26.1%)	25 (17.9%)	74 (43.5%)	
Moderate	571 (47.7%)	430 (42.3%)	82 (59.4%)	94 (67.1%)	81 (47.6%)	

High	68 (5.7%)	21 (2.1%)	17 (12.3%)	17 (12.1%)	9 (5.3%)	
Low-salt and low-fat diet, n (%)	405 (33.8%)	283 (27.9%) *	39 (28.3%)	21 (15.0%) ***	61 (35.9%)	< 0.001
Regular exercise, n (%)	343 (28.6%)	313 (30.8%)	57 (41.3%) **	28 (20.0%) *	50 (29.4%)	< 0.05
Hypotensive medication, n (%)	283 (23.6%)	306 (30.1%) **	28 (20.3%)	33 (23.6%)	41 (24.1%)	< 0.05
Family history of diabetes, n (%)	197 (16.4%)	195 (19.2%)	21 (15.2%)	10 (7.1%) **	35 (20.6%)	< 0.01
Current smoker, n (%)	140 (11.7%)	266 (26.2%) ***	22 (15.9%)	14 (10.0%)	24 (14.1%)	< 0.001
Habitual alcohol drinker, n (%)	178 (14.9%)	344 (33.9%) ***	20 (14.5%)	17 (12.1%)	33 (19.4%)	< 0.001

Abbreviations: BMI: body mass index, WC: waist circumference, SBP: systolic blood pressure, DBP: diastolic blood pressure, FPG: fasting plasma glucose, HbA1c: hemoglobin A1c, TC: total cholesterol, TG: triglyceride, LDL-C: low-density lipoprotein cholesterol, HDL-C: high-density lipoprotein cholesterol, eGFR: estimated glomerular filtration rate.
Data are presented as means ± standard deviations or numbers (%), where appropriate. One-way analysis of variance was used for comparisons of continuous variables, while the $\chi 2$ test was used for comparison of categorical variables.
Post hoc comparisons were adjusted using Bonferroni's correction.
*p < 0.05 and **p < 0.01, ***p < 0.001, compared to participants with non-tea consumer.

Supplementary Table S2. Subgroup analysis in male and female participants.

	Male					Female				
		Model 1		Model 2			Model 1		Model 2	
	N, cases/	OR (95% CI)	P	OR (95% CI)	P	N, cases/	OR (95% CI)	P	OR (95% CI)	P
Progression to diabetes										
Type of tea										
No tea	36/282	1.00 (Ref.)		1.00 (Ref.)		90/916	1.00 (Ref.)		1.00 (Ref.)	
Green tea	88/509	1.30 (0.84, 2.00)	0.24	1.25 (0.80, 1.96)	0.33	53/507	0.99 (0.69, 1.43)	0.96	1.03 (0.70, 1.51)	0.89
Black tea	6/58	0.76 (0.30, 1.93)	0.56	0.97 (0.37, 2.57)	0.96	3/80	0.36 (0.11, 1.18)	0.09	0.32 (0.09, 1.07)	0.07
Dark tea	2/41	0.34 (0.08, 1.48)	0.15	0.34 (0.07, 1.54)	0.16	3/99	0.27 (0.08, 0.89)	0.03	0.27 (0.08, 0.90)	0.03
Other	6/66	0.73 (0.29, 1.87)	0.52	0.72 (0.27, 1.88)	0.50	12/104	1.37 (0.71, 2.66)	0.35	1.49 (0.75, 2.96)	0.26
Regression to normoglycemia										
Type of tea										
No tea	83/282	1.00 (Ref.)		1.00 (Ref.)		261/916	1.00 (Ref.)		1.00 (Ref.)	
Green tea	114/509	0.73 (0.52, 1.03)	0.07	0.74 (0.52, 1.06)	0.10	118/507	0.76 (0.59, 0.98)	0.04	0.76 (0.58, 0.99)	0.04
Black tea	16/58	0.87 (0.46, 1.66)	0.68	1.00 (0.51, 1.99)	0.99	25/80	1.04 (0.63, 1.71)	0.88	1.00 (0.59, 1.71)	0.99
Dark tea	12/41	0.87 (0.42, 1.81)	0.72	1.36 (0.61, 3.02)	0.45	27/99	0.85 (0.53, 1.35)	0.49	0.63 (0.37, 1.05)	0.07
Other	23/66	1.22 (0.68, 2.19)	0.50	1.28 (0.69, 2.38)	0.43	37/104	1.46 (0.94, 2.27)	0.10	1.16 (0.73, 1.85)	0.53

Multinominal logistic regression analysis was employed to assess the associations of tea consumption with prediabetes progression and prediabetes regression.
Model 1: The crude model.
Model 2: Adjusted for age, body mass index, mean arterial pressure, triglyceride, high-density lipoprotein-cholesterol, low-density lipoprotein-cholesterol, estimated glomerular filtration rate, low salt low-fat diet, regular exercise, Hypotensive medication, family history of diabetes, current smoking status, current alcohol consumption, vegetable consumption, and fruit consumption.

Supplementary Table S3. Subgroup analysis in participants with Han and other ethnicity.

	Han ethnicity					Other ethnicity				
	N, cases/ total	Model 1		Model 2		N, cases/ total	Model 1		Model 2	
		OR (95% CI)	P	OR (95% CI)	P		OR (95% CI)	P	OR (95% CI)	P
Progression to diabetes										
Type of tea										
No tea	114/1024	1.00 (Ref.)		1.00 (Ref.)		12/174	1.00 (Ref.)		1.00 (Ref.)	
Green tea	135/954	1.20 (0.91, 1.58)	0.19	1.05 (0.78, 1.42)	0.74	6/62	1.34 (0.48, 3.80)	0.58	1.23 (0.38, 3.97)	0.73
Black tea	3/40	0.70 (0.21, 2.35)	0.56	0.64 (0.19, 2.20)	0.48	6/98	0.93 (0.33, 2.60)	0.89	0.42 (0.12, 1.49)	0.18
Dark tea	2/10	2.13 (0.41, 11.11)	0.37	2.19 (0.40, 12.13)	0.37	3/130	0.33 (0.09, 1.21)	0.10	0.18 (0.04, 0.80)	0.02
Other	17/149	1.16 (0.66, 2.04)	0.60	1.05 (0.59, 1.88)	0.87	1/21	0.72 (0.09, 5.96)	0.76	0.86 (0.09, 7.86)	0.89
Regression to normoglycemia										
Type of tea										
No tea	303/1024	1.00 (Ref.)		1.00 (Ref.)		41/174	1.00 (Ref.)		1.00 (Ref.)	
Green tea	221/954	0.74 (0.60, 0.91)	0.01	0.72 (0.58, 0.90)	0.01	11/62	0.72 (0.34, 1.53)	0.39	0.65 (0.29, 1.46)	0.30
Black tea	14/40	1.22 (0.62, 2.40)	0.57	1.16 (0.57, 2.33)	0.69	27/98	1.23 (0.69, 2.17)	0.49	1.14 (0.57, 2.29)	0.72
Dark tea	3/10	1.20 (0.29, 5.06)	0.80	0.83 (0.19, 3.61)	0.80	36/130	1.17 (0.69, 1.97)	0.56	0.99 (0.53, 1.86)	0.98
Other	54/149	1.39 (0.96, 2.02)	0.09	1.21 (0.82, 1.79)	0.34	6/21	1.27 (0.46, 3.51)	0.65	0.96 (0.33, 2.81)	0.94

Multinominal logistic regression analysis was employed to assess the associations of tea consumption with prediabetes progression and prediabetes regression.

Model 1: The crude model.

Model 2: Adjusted for age, sex, body mass index, mean arterial pressure, triglyceride, high-density lipoprotein-cholesterol, low-density lipoprotein-cholesterol, estimated glomerular filtration rate, low salt low-fat diet, regular exercise, Hypotensive medication, family history of diabetes, current smoking status, current alcohol consumption, vegetable consumption, and fruit consumption.
